# Ectopic meningioma in a patient with neurofibromatosis Type 2: a case report and review of the literature

**DOI:** 10.1259/bjrcr.20180007

**Published:** 2018-04-23

**Authors:** Chun Qiu Su, Shan Shan Lu, Mao Dong Zhou, Xun Ning Hong

**Affiliations:** 1 Department of Radiology,The First Affiliated Hospital of Nanjing Medical University,Nanjing,China

## Abstract

Ectopic meningioma occurring in the region of parapharyngeal space is rare in clinical practice and brings great challenge in its diagnosis. This report details such a case in a 14-year-old girl with neurofibromatosis Type 2, which is a highly infrequent association. The clinical manifestations, imaging findings, and pathological manifestations are described, and the relevant literature is reviewed to highlight characteristic imaging findings of ectopic meningiomas.

## BACKGROUND

Meningiomas are among the most common tumours of the central nervous system, accounting for nearly 20% of primary intracranial tumours.^1^ Meningiomas can exist as intra- or extracranial brain tumours; however, extracranial meningiomas, also known as ectopic meningiomas, are extremely rare. They have been reported to occur at various anatomic sites, including the orbit,^2^ middle ear,^3^ tongue,^4^ mediastinum,^5^ and fingers.^6^ The diagnosis may be especially challenging when such unusual anatomic sites are involved. Neurofibromatosis Type 2 (NF2) is a genetic neoplastic disorder that presents with multiple meningiomas and bilateral vestibular schwannomas,^7, 8^ and most meningiomas associated with NF2 arise intracranially. Herein, we report the clinical signs, imaging findings, and pathological manifestations of a rare case of meningioma occupying the parapharyngeal space in a patient with NF2.

## CASE REPORT

**Figure 1.**
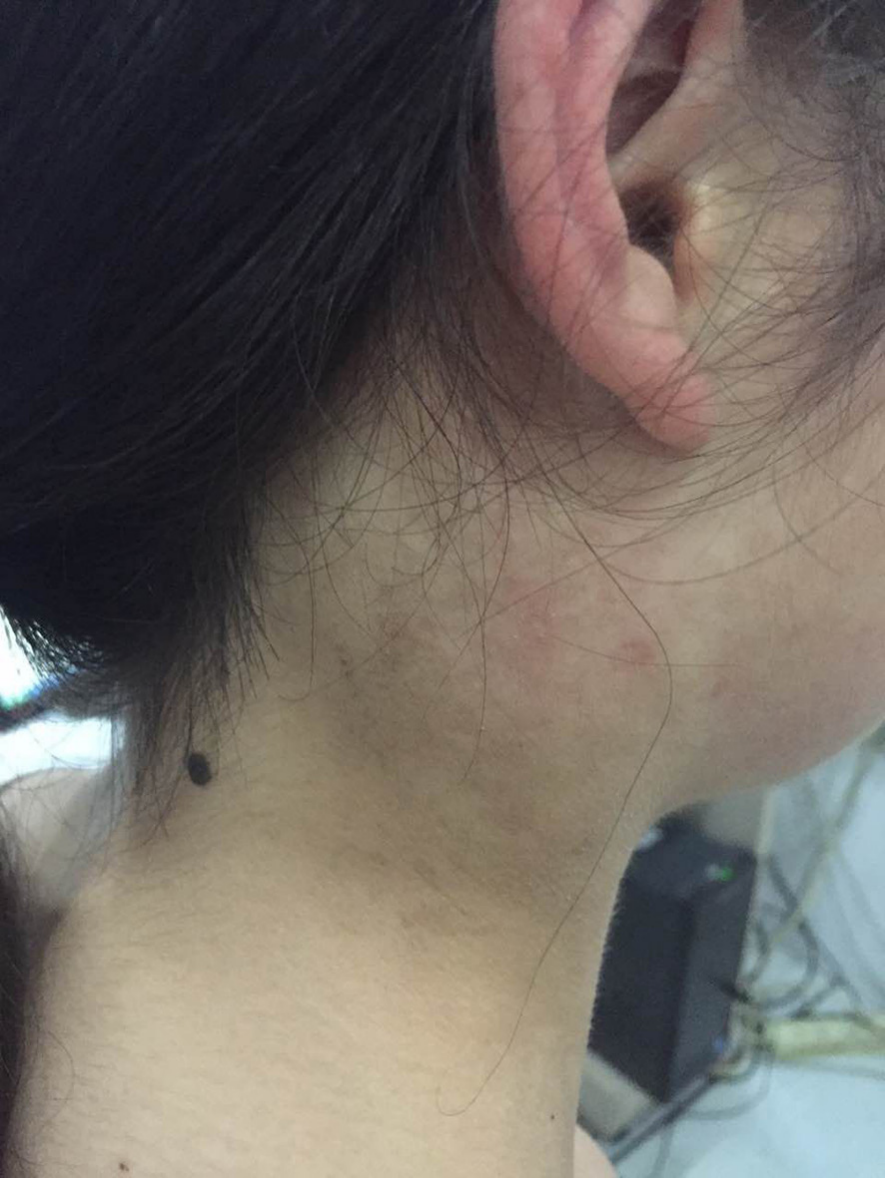
The patient, a 14-year-old girl, presented with a painless mass in the right retroauricular area.

Our patient, a 14-year-old girl, presented with a 4 month history of a painless mass in the right retroauricular area; it was first found in June 2015. Clinical examination revealed a mass approximately 4 × 3 cm in size (Figure 1). The neurological examination was negative. The patient’s past medical history and her family’s medical history were unremarkable.

**Figure 2.**
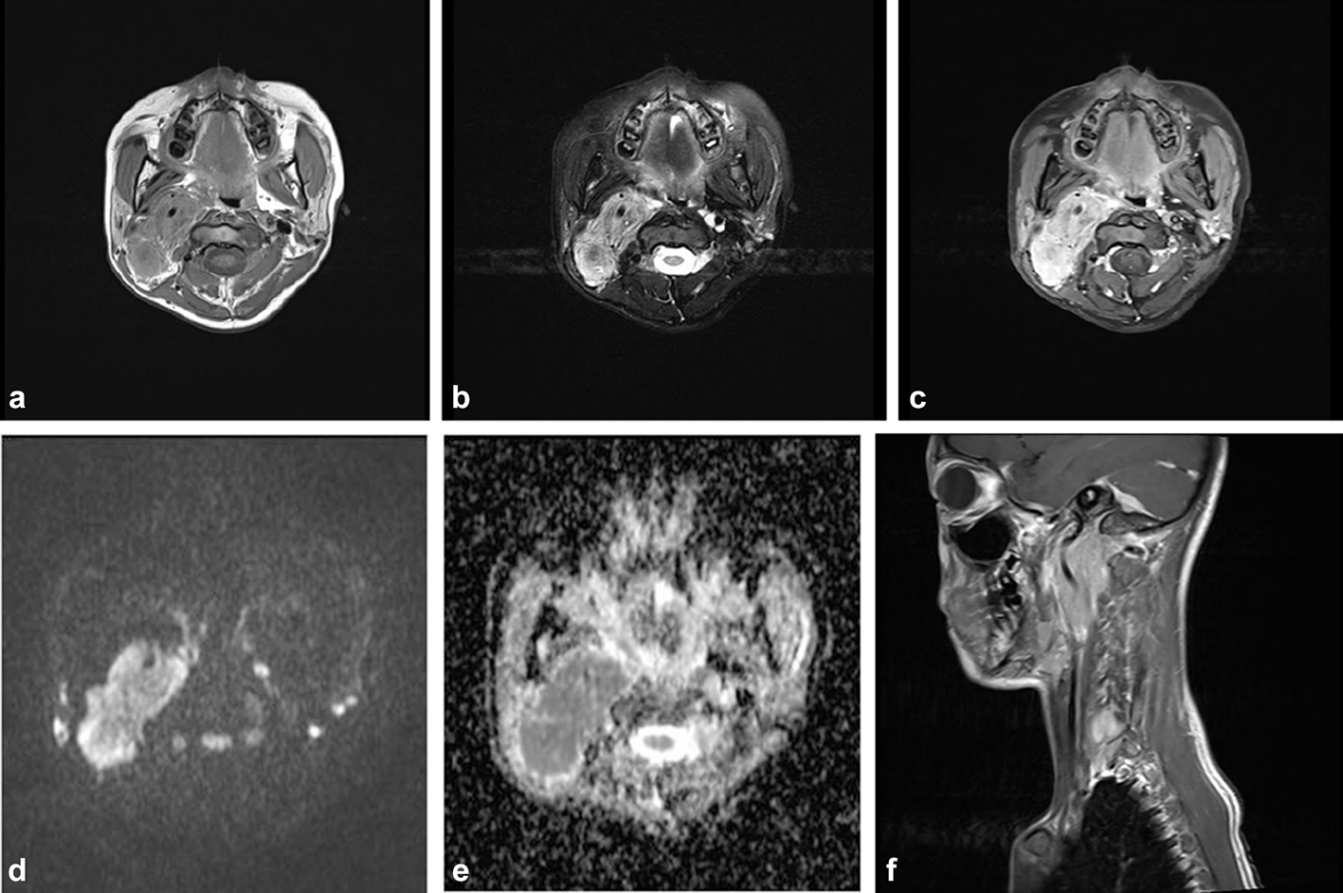
Nasopharyngeal MRI examination reveals a large, well-circumscribed mass in the right parapharyngeal space. The mass is isohypointense on *T*_1_WI (a) and slightly hyperintense on *T*_2_WI (b); it is accompanied by multiple focal hypointensities that may represent calcification. Marked but heterogeneous enhancement was observed on axial post-contrast *T*_1_WI after the administration of gadolinium (c). The sagittal post-contrast *T*_1_WI (f) shows that the mass had invaded the carotid space—as well as the parotid and perivertebral space—and encased major carotid vessels. DWI (d) revealed a restriction of diffusion, with an average ADC value of 699.7 × 10^−6^ mm^2^ s^–^^1^ (e). ADC, apparent diffusion coefficient; DWI, diffusion-weighted imaging; *T*_1_WI,*T*_1 _weighted imaging; *T*_2_WI,*T*_2 _weighted imaging.

A nasopharyngeal MRI examination revealed a well-circumscribed mass in the right parapharyngeal space that measured 5.4 × 2.4 × 5.5 cm. The mass had invaded the carotid space as well as the parotid and perivertebral space—and encased major carotid vessels. It was isohypointense on *T*_1_ weighted imaging (*T*_1_WI) and slightly hyperintense on *T*_2_ weighted imaging (*T*_2_WI) and accompanied by multiple focal hypointensities that might have suggested calcification. Marked but heterogeneous enhancement was observed after the administration of gadolinium. Diffusion-weighted imaging revealed diffusion restriction; the average apparent diffusion coefficient  value was 699.7 × 10^−6^ mm^2^ s^–1^ (Figure 2).

**Figure 3.**
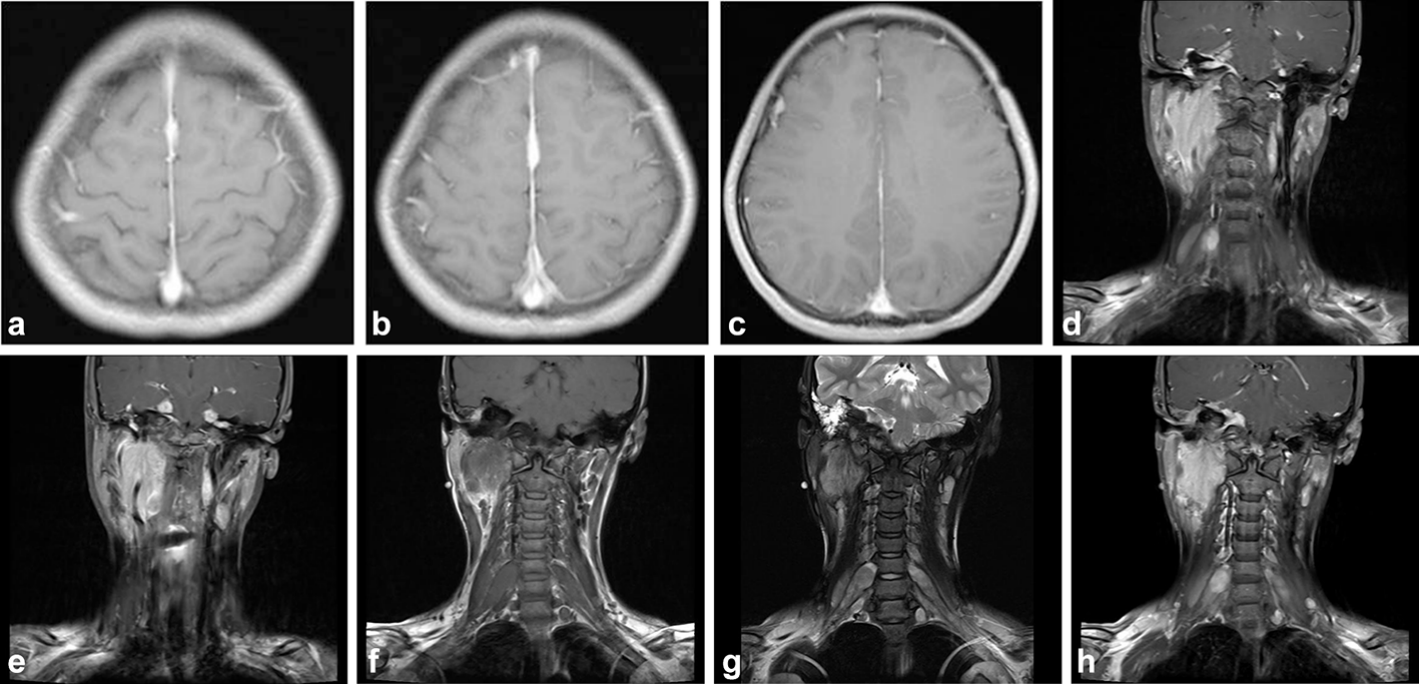
An axial post-contrast *T*_1_WI (a–c), reveals multiple nodular masses in the frontal region and the midline (black arrows). There were bilateral masses in the bilateral cerebellopontine angle, which broadened bilateral internal auditory canal. They showed significant contrast enhancement after gadolinium administration (white arrow). There were bilateral thickening C6-C7 spinal nerve root. These roots were isointense on both coronal *T*_1_WI (f) and *T*_2_WI (g). In addition, obvious nodular enhancement is seen on the coronal post-contrast *T*_1_WI (h) (red arrows). The coronal post-contrast *T*_1_WI shows involvement of the right hypoglossal canal (yellow arrows). *T*_1_WI,*T*_1 _weighted imaging; *T*_2_WI,*T*_2 _weighted imaging.

Imaging revealed multiple intracranial meningiomas, bilateral schwannomas in the cerebellopontine angle, and multiple neurofibromas at the C6-C7 spinal nerve root; according to the accepted diagnostic criteria, these were compatible with NF2.^8^ The coronal post-contrast *T*_1_WI revealed involvement of the right hypoglossal canal (Figure 3).

**Figure 4.**
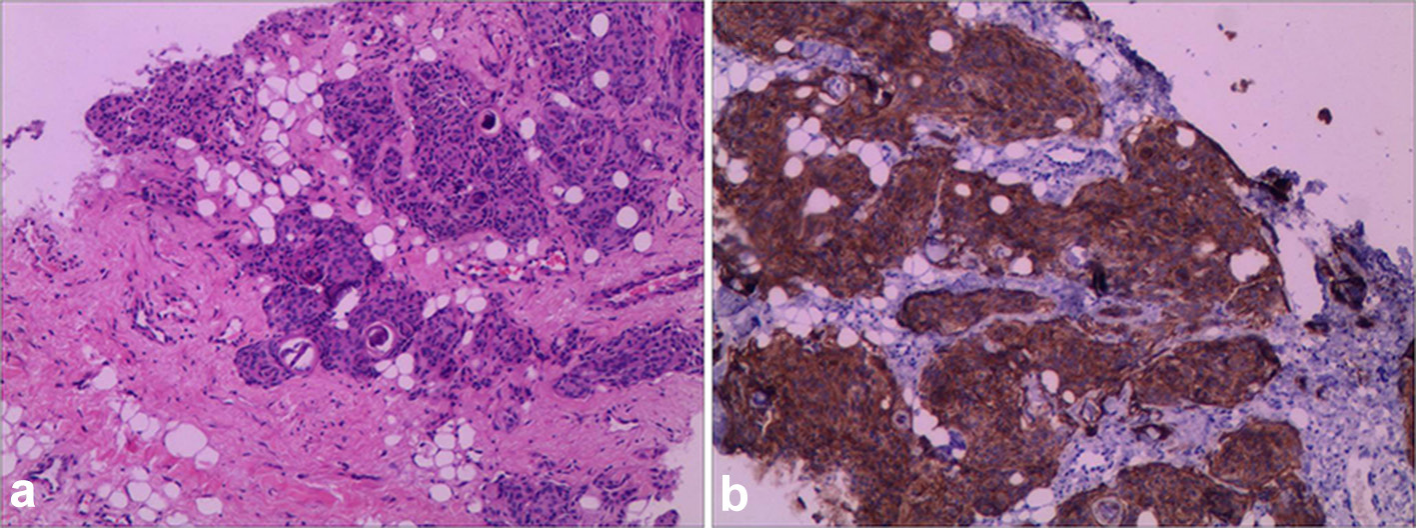
(a) H-E staining shows psamomma bodies in sheets of cells with a meningothelial pattern (H-E;×200). (b) Immunohistochemistry shows positive staining for EMA. (Magnification × 100). EMA, epithelial membrane antigen; H-E, haematoxylin and eosin.

The patient underwent a needle puncture biopsy of the mass in June 2015. Haematoxylin and eosin (H-E) staining revealed that there was a heavy deposit of collagen fibers between the tumour cells; multiple psammoma bodies were also seen. The tumour itself consisted mainly of bundles of elongated spindle-shaped cells with small oval nuclei. The typical whorl structure of meningioma was observed in some areas. Immunohistochemical staining showed that the tumour cells were positive for progesterone receptor, epithelial membrane antigen, and vimentin and negative for S-100 protein, CK, CD68, and P53. The Ki-67 labelling index was less than 1%(Figure 4). In view of the H-E staining and immunohistochemical findings, the tumour was diagnosed as a benign ectopic meningioma of World Health Organization Grade I based on the 2007 World Health Organization classification.^9^ In light of her young age and the absence of symptoms, the neurosurgeons followed her annually until she reached 20 years of age. This was considered preferable to receiving the surgery and radiation therapy. At the latest follow-up of 2.5 years, the patient had no new complaints and the mass had remained of almost the same size.

## DISCUSSION

 Extracranial meningiomas are extremely rare, with an overall incidence of 1–2%.^1^ Four hypotheses regarding the development of ectopic meningiomas have been proposed; that they arise due to (1) metastasis from an intracranial meningioma, (2) direct extension from an intracranial lesion, (3) growth from embryonic nests of arachnoid cells, and (4) the development of arachnoid cells within cranial nerve sheaths.^10^ According to these hypotheses, our case may have arisen from the cranial nerves with extracranial parapharyngeal extension. It has also been suggested that primary ectopic meningiomas of the head and neck are related to neurofibromatosis, particularly NF2. NF2 is caused by a mutation in a tumour suppressor gene on chromosome 22q12 and is characterized by vestibular schwannomas and meningiomas.^11^

The diagnosis of ectopic meningioma is difficult to make on the basis of imaging alone. On MRI, meningiomas usually have homogenous contrast enhancement and signal intensity similar to that of other brain lesions. The diagnosis of ectopic meningioma in the parapharyngeal space is particularly challenging, since the more common tumours occurring in this anatomical space include neurogenic tumours and paragangliomas. Often, the dural tail component, intracranial extension, and demonstration of calcification should prompt the clinician to consider the possibility of meningioma.^12^ The dural tail sign is, however, not detected outside of the brain, in our case it could not aid in the diagnosis. We did find multiple focal hypointensities on *T*_1_WI and *T*_2_WI, which might have suggested calcification. MRI is an essential diagnostic tool for the identification of parapharyngeal masses and can reflect the extent of the tumour. These imaging modalities are also extremely useful in pre-operative surgical planning.

A definitive diagnosis depends on histopathological and immunohistochemical findings. It is axiomatic that the histopathological and immunohistochemical findings for ectopic meningioma are similar to those for other intracranial lesions. In this case, H-E staining revealed that the tumour consisted of collagen fibres and elongated spindle cells, which are common features of meningioma.^1, 5^ The finding of multiple psammoma bodies and typical whorl formations further confirmed the diagnosis of meningioma.^1, 5^ Immunohistochemistry is an essential tool for confirming the diagnosis of extracranial meningioma. The tumour cells in our report showed positive staining for epithelial membrane antigen and vimentin, which further supported the diagnosis.

Although the current standard of care for meningiomas is surgery, NF2-associated meningiomas and all developing masses can be resected by stereotactic radiosurgery, a non-invasive form treatment.^8^ However, decisions regarding therapy in cases such as ours must be made on an individual basis. Previous studies have reported that meningioma is a marker of disease severity in NF2, being associated with a 2.5- fold greater risk of mortality as compared with NF2 patients without meningiomas.^13^ The patient in our case was not offered treatment because, after 2 years of follow-up, she had no new symptoms and the mass was still of almost the same size. In a word, the prognosis of ectopic meningioma appears to be good, with an overall median survival of 28 years. However, in any individual case, an estimate of survival is decided by the tumour’s histological type, anatomic site, and grade as well as the patient’s age.^1^

## LEARNING POINTS

1. The possibility of an ectopic meningioma should be considered if a lesion is found in the parapharyngeal space of a patient with NF2.
2. The imaging and histopathological findings of an ectopic meningioma in the parapharyngeal space are indistinguishable from the findings of other intracranial lesions.
3. MRI plays an important role in the diagnosis. This imaging modality is also extremely useful in surgical planning and subsequent follow-up.
